# Air Pollution And Cardiovascular Risk Factors In Older Adults: Evidence From The Lookup 8+ Project

**DOI:** 10.1093/geroni/igaf122.4066

**Published:** 2025-12-31

**Authors:** Matteo Tosato, Riccardo Calvani, Stefano Cacciatore, Emanuele Marzetti, Francesco Landi

**Affiliations:** Fondazione Policlinico Universitario Agostino Gemelli IRCCS, Rome, Lazio, Italy; Università Cattolica del Sacro Cuore, Rome, Lazio, Italy; Fondazione Policlinico Universitario Gemelli IRCCS, Rome, Lazio, Italy; Università Cattolica del Sacro Cuore, Rome, Lazio, Italy; Università Cattolica del Sacro Cuore, Rome, Lazio, Italy

## Abstract

Air pollution is increasingly recognized as an important determinant of cardiovascular health, yet evidence in older adults remains limited. We analyzed data from 6077 community-dwelling participants of the Longevity Check-up (Lookup) 8+ cohort across 22 Italian cities spanning mainland Italy and major islands to examine the association between PM2.5 exposure and clinical risk factors. Annual mean PM2.5 concentrations were estimated by integrating ground monitoring station and satellite-based data. Exposure was calculated at the municipality level and restricted to participants residing in urban areas. Participants were stratified by exposure above or below the regulatory threshold of 25 µg/m³. The study sample had a mean age of 70.4 years (53.6% women, BMI 26.0 kg/m²). Of these, 1538 (25.3%) were exposed to PM2.5 ≥25 µg/m³. Compared with those below threshold, highly exposed participants showed significantly higher systolic blood pressure (134.7 vs 128.1 mmHg; +6.5 mmHg, p < 0.001), diastolic pressure (77.6 vs 75.6 mmHg; +2 mmHg; p < 0.001), and blood cholesterol (213.7 vs 199.8 mg/dL; +13.9 mg/dL; p < 0.001). Linear regression analysis confirmed a quantitative dose-response with each 1 µg/m³ increase in PM2.5 exposure being associated with +0.45 mmHg systolic blood pressure and +1.2 mg/dL cholesterol. Associations remained statistically significant after adjustment for major confounders including age, sex, BMI, smoking status, diabetes, and physical activity. These findings provide evidence linking PM2.5 exposure to adverse cardiovascular profiles in older adults and highlight the importance of considering environmental determinants in geriatric healthcare.

